# The Hidden Fortress: A Comprehensive Review of Fungal Biofilms with Emphasis on *Cryptococcus neoformans*

**DOI:** 10.3390/jof11030236

**Published:** 2025-03-19

**Authors:** Hope M. Pruitt, Jinyi C. Zhu, Sean P. Riley, Meiqing Shi

**Affiliations:** Department of Veterinary Medicine, Virginia-Maryland College of Veterinary Medicine, University of Maryland, College Park, MD 20742, USA; hpruitt@umd.edu (H.M.P.); cjzhu777@umd.edu (J.C.Z.)

**Keywords:** fungus, biofilm, *Cryptococcus neoformans*, *Candida*, *Aspergillus*, *Trichosporon*, *Fusarium*, *Coccidioides*, cryptococcosis, virulence factors, polysaccharide capsule, meningoencephalitis, titan cell, extracellular matrix, central nervous system

## Abstract

Biofilms are structurally organized communities of microorganisms that adhere to a variety of surfaces. These communities produce protective matrices consisting of polymeric polysaccharides, proteins, nucleic acids, and/or lipids that promote shared resistance to various environmental threats, including chemical, antibiotic, and immune insults. While algal and bacterial biofilms are more apparent in the scientific zeitgeist, many fungal pathogens also form biofilms. These surprisingly common biofilms are morphologically distinct from the multicellular molds and mushrooms normally associated with fungi and are instead an assemblage of single-celled organisms. As a collection of yeast and filamentous cells cloaked in an extracellular matrix, fungal biofilms are an extreme threat to public health, especially in conjunction with surgical implants. The encapsulated yeast, *Cryptococcus neoformans,* is an opportunistic pathogen that causes both pulmonary and disseminated infections, particularly in immunocompromised individuals. However, there is an emerging trend of cryptococcosis among otherwise healthy individuals. *C. neoformans* forms biofilms in diverse environments, including within human hosts. Notably, biofilm association correlates with increased expression of multiple virulence factors and increased resistance to both host defenses and antifungal treatments. Thus, it is crucial to develop novel strategies to combat fungal biofilms. In this review, we discuss the development and treatment of fungal biofilms, with a particular focus on *C. neoformans*.

## 1. Introduction

The term “biofilm” has been used for nearly 40 years to describe communities of microorganisms encased in extracellular matrices—a concept first observed by van Leeuwenhoek and Pasteur [1,2,3]. Biofilm-associated organisms enhance immune evasion, antimicrobial resistance, and physical resilience, driving research into their mechanisms and pathogenicity. Biofilms are structured microbial communities attached to surfaces and embedded within an extracellular matrix (ECM) [4,5]. Each organism produces a unique ECM composed of a combination of polysaccharides, proteins, nucleic acids, and/or lipids, creating a protective and adaptive environment [4]. Water channels within mature biofilms facilitate nutrient distribution and waste removal, supporting embedded microbes [6,7,8]. Biofilm-associated organisms exhibit distinct transcriptional profiles, altered growth rates, quorum sensing, and polymer secretion [7,9,10,11,12]. Together, this complex structure shields the densely packed microbes from chemical disruption, antimicrobial damage, and phagocytosis [9,13].

More than 65% of human microbial infections involve biofilms [14], with bacterial biofilms primarily studied due to their more readily observable role in nature [10,15,16]. However, interest in fungal biofilms has expanded in tandem with the rise in clinical cases involving these networks. Various fungal pathogens possess the ability to generate biofilms with unique properties impacting public health [11,17,18,19,20,21,22]. A foundational study by Murillo et al. on the fungal pathogen *Candida albicans* revealed differences in gene expression between planktonic and biofilm states, with clear differences in metabolism, stress response, and cell wall biosynthesis—key factors in pathogenicity and antimicrobial resistance [13,16]. Fungal biofilms exhibit significantly higher resistance to antifungal treatments than their planktonic counterparts, contributing to persistent infections [23,24,25,26].

These principle characteristics of fungal biofilms have also been investigated in pathogenic *Cryptococcus* spp., where cells within biofilms demonstrate elevated levels of proteins associated with oxidative reduction, proteolysis, and stress responses while showing reduced levels of proteins involved in metabolism, transport, and translation [27]. In addition, *C. neoformans* readily adheres to various surfaces, including living tissues and medical devices [10]. Therefore, as the prevalence of implanted medical objects like, shunts and catheters, has increased, so too has the frequency of medically-relevant cryptococcal biofilms. This surge in cases has raised significant concerns within the scientific and medical communities, as there remains a great deal of uncertainty surrounding biofilms in general, particularly those related to fungi.

Several other fungal species also form biofilms or organize into communities that demonstrate biofilm-like characteristics. These biofilms are highly prevalent in natural environments; however, certain species can also act as opportunistic pathogens in clinical settings [28,29,30]. Examples of these pathogenic fungi include species within the genera *Candida*, *Aspergillus*, *Malassezia*, *Trichosporon*, *Fusarium*, and *Coccidioides* [17,18,22,23,25,31,32]. While some of these species are considered part of the normal mammalian flora, all can become pathogenic under certain conditions [33,34,35,36]. The biofilms from each of these pathogens display similar general architecture and components. However, the characteristics of biofilms vary significantly across species and environmental conditions [4,37,38]. Infections caused by biofilm-associated fungal pathogens, particularly *Cryptococcus*, *Candida*, and *Aspergillus* species, are estimated to cause over one million deaths annually, emphasizing the urgent need for a deeper understanding of biofilm-associated pathogenesis [39]. In this review, our primary focus is on *C. neoformans* pathogenesis and biofilm formation. However, we also highlight the key characteristics of medically relevant fungal biofilms and summarize current therapeutic approaches and ongoing research efforts designed to combat fungal biofilms.

## 2. *Cryptococcus* Biology and Its Role in Biofilm Development

*Cryptococcus* infection, or cryptococcosis, is a fungal infection that most commonly affects immunocompromised individuals. While the fungus initially infects the lung it can occasionally disseminate to cause life-threatening conditions such as meningitis. The two *Cryptococcus* species most associated with clinically relevant human infections are *C. neoformans* and *C. gattii*. *C. neoformans* is the most widely studied *Cryptococcus* species and, therefore, will be the primary topic of this review. *C. neoformans* was first characterized as a mammalian pathogen in the late 19th century and was recognized as a cause of human disease early in the 20th century [40,41]. Its significance as an opportunistic pathogen, particularly in immunocompromised individuals, became more evident with the advent of immunosuppressive therapies and later during the human immunodeficiency virus/acquired immune deficiency syndrome (HIV/AIDS) epidemic [41].

Metabolically active *Cryptococcus* spp. exist as basidiomycete yeasts that commonly divide by budding but can perform mating in conjunction with short hyphae formation and spore generation [41]. In certain environments, *Cryptococcus* can also expand to form polyploid titan cells and assemble into biofilms [11,42,43,44,45,46]. *C. neoformans* cells are commonly divided into several serotypes based on agglutination reactions of the capsular components, including serotypes A, D, and hybrid AD. *C. neoformans* serotype A strain is the most prevalent serotype, accounting for approximately 95% of all cryptococcal infections [41]. With the increase in susceptible human populations, changes to the distribution of infectious agents, and the significant public health burden, scientists are now placing greater emphasis on understanding the basic biology and pathobiology of infectious *Cryptococcus* spp.

*Cryptococcus* species form biofilms both in natural environments and within host tissues, contributing to survival and pathogenicity [47,48]. In nature, these biofilms develop on surfaces such as plant matter and soil, where they provide protection against environmental stresses and/or predation [47,49]. Biofilms also aid in nutrient acquisition by concentrating metabolic activity and enabling cooperative interactions among fungal cells [15,50]. In vivo, cryptococcal biofilms form on medical devices and within tissues, enhancing resistance to antifungal treatments and facilitating persistence within the host [42,51]. Given its role in both environmental survival and host persistence, understanding the formation and regulation of biofilms in *Cryptococcus* species is crucial for improving our management of cryptococcosis.

### 2.1. Dynamics of Cryptococcal Infection

#### 2.1.1. Cryptococcosis: Infection and Latency

*C. neoformans* yeasts and spores are ubiquitous in soil, bird excreta, decaying organic material, and certain types of trees [52,53]. Fungal spores are released into the air and are readily inhaled by humans and animals because the 1.5–3.5 µm *Cryptococcus* spores are small enough to easily travel to the lungs, whereas particles larger than 5 µm are efficiently cleared by the mucociliary airway [53,54]. Therefore, cryptococcosis invariably begins as a pulmonary infection. In healthy hosts, this infection is most commonly asymptomatic or presents as cold-like symptoms that subside with or without antifungal therapy [55,56]. However, individuals with weakened immune systems are susceptible to persistent lung infection or dissemination from the lungs to the central nervous system. This disseminated infection is known as cryptococcal meningoencephalitis/meningitis [57]. This condition is of particular interest, as *Cryptococcus* is a leading cause of adult meningitis in numerous regions across the globe [57].

As a spore-forming fungus, *C. neoformans* can become dormant within the body, resulting in latent infection [58]. This is evidenced by the capacity of fungal spores to survive within phagocytes without inducing an effective intracellular innate immune response [59]. In the absence of intracellular killing of spores, the immune system often forms granulomas in an attempt to cordon off the infected myeloid cells [60]. These granulomas are highly structured, with a core of either actively or latently infected macrophages surrounded by layers of additional immune cells, including lymphocytes and fibroblasts [61]. Latent *C. neoformans* can reemerge from these granulomas to reinitiate fulminant infection when the host experiences an immunosuppressed environment, such as the development of chronic diseases, age-related issues, AIDS, and immunosuppressive medications [62,63,64]. Thus, there are two sources of severe cryptococcosis: primary infection and latent infection. Each of these sources of infection can result in systemic fungal infection in an immunocompromised host.

#### 2.1.2. Cryptococcosis: Epidemiology, Clinical Presentation, and Treatment

Cryptococcosis has a worldwide environmental distribution and is associated with a wide variety of clinical symptoms that depend on both the pathogenicity of the fungus and the immune status of the host. Serological investigations demonstrate that exposure to *Cryptococcus* in humans is extremely prevalent, but fulminant disease development is comparatively rare [65]. Until the early 1980s, cryptococcosis was considered a rare disease that primarily affected immunocompromised individuals. However, the HIV/AIDS epidemic dramatically shifted this perspective, as cryptococcosis became a leading cause of death among patients suffering from this condition. By the mid-1980s, the prevalence of cryptococcosis had increased significantly, where 80% of *Cryptococcus* infections involved patients with HIV/AIDS [63,66]. It was soon discovered that the acute susceptibility of AIDS patients to cryptococcosis was a result of severely impaired CD4^+^ T-cell immunity [67].

Fortunately, the successful and widespread implementation of anti-retroviral therapy (ART) has greatly decreased the incidence of AIDS andconsequently decreased the burden of HIV-associated cryptococcosis. However, these positive developments have mainly occurred in developed nations with access to the necessary medications. As such, HIV-associated cryptococcosis remains prevalent in developing regions, specifically in sub-Saharan Africa, where there is a higher number of at-risk populations [68]. While ART has been a groundbreaking advancement for preventing the rapid progression of AIDS, another somewhat surprising *Cryptococcus*-associated condition has recently been identified that is a direct consequence of the re-emergence of immunocompetence after ART therapy. Immune reconstitution inflammatory syndrome (IRIS), occurs in 10% to 45% of AIDS patients who are actively or latently infected with *C. neoformans*. In these cases, the rapid reemergence of *C. neoformans*-specific CD4^+^ T-cells in conjunction with existing infection induces widespread disseminated inflammation, with severe consequences for the patient [69].

Although cryptococcosis initially manifests as a pulmonary infection, its progression to the central nervous system (CNS) represents the most severe and clinically significant form of the disease [57]. Dissemination of *C. neoformans* into the CNS results in an infection called cryptococcal meningitis (CM), and this infection is associated with a rapid escalation in clinical signs and symptoms. CM is a severe and often fatal condition, with approximately 152,000 annual cases and a 74% case fatality rate [70]. CM patients present with diverse neurological symptoms, such as headache, altered mental status, stiff neck, lethargy, fever, nausea, and vomiting [71,72]. More rare manifestations include dementia, seizures, hearing loss, and vision disturbances [71]. Due to the similarity of CM symptoms to various other conditions and low clinical suspicion, diagnosis of CM is frequently missed or delayed. Therefore, the World Health Organization and the Infectious Diseases Society of America recommend conducting a lumbar puncture followed by a rapid cryptococcal antigen assay of the cerebrospinal fluid in patients presenting with certain pulmonary and neurological symptoms. If this assay is unavailable, an India ink microscopy test is advised [73,74]. Screening for plasma, serum, or whole-blood cryptococcal antigen is the optimal approach for guiding resources in a public health approach. This is especially imperative when considering those who are living with HIV [73].

The United States Centers for Disease Control and Prevention recommends that all individuals diagnosed with cryptococcosis should take antifungal medications for at least six months. Fluconazole is recommended for mild to moderate lung infections as well as for asymptomatic infections in those who are HIV/AIDS positive [75]. Initial treatment of severe lung infections and/or CM requires liposomal amphotericin B (AmB) and flucytosine, followed by fluconazole [75]. The regimen for AIDS-positive patients is significantly different from that for immunocompetent patients. In these cases, CM requires a multistep management plan that begins with an intensive antifungal regimen, known as the induction phase. To prevent IRIS, antiretroviral therapy is only started after this aggressive antifungal therapy.. The clinical treatment regimen then progresses through additional consolidation and maintenance phases, with a gradual adjustment in antifungal therapy [75]. Effective management of cryptococcosis requires timely diagnosis, appropriate antifungal therapy, and careful coordination with ART initiation, underscoring the importance of an integrated approach to reducing the global burden of this devastating disease.

### 2.2. Virulence Factors and Immune Evasion Strategies of C. neoformans

#### 2.2.1. Virulence-Associated Structural Components of Yeast Cell

**Figure 1:**
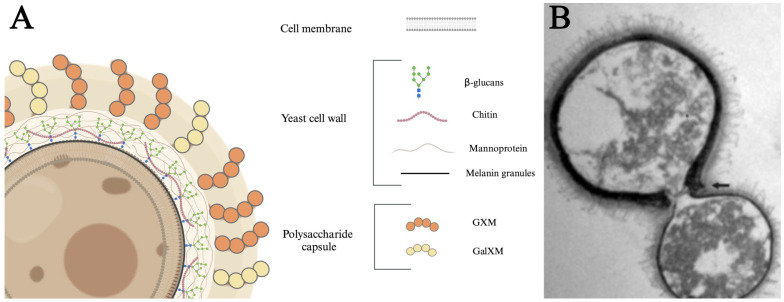
Structure and components of *C. neoformans.* (**A**) The proximal cell membrane consists of a phospholipid bilayer composed of various proteins and lipids. The central cell wall consists of a dense conglomeration of β-glucans, mannoproteins, chitin, and melanin. The distal layer consists of a 5–10 µm capsule primarily made of glucuronoxylomannan (GXM) and galactoxylomannan (GalXM). (**B**) Transmission electron micrograph of a mother cell with a clear dark melanin-containing cell wall and a budding daughter cell. The arrow indicates the disrupted edges of the mother cell. The dark pigmentation is the melanin granules and the fibrillar structures on the cell surface are polysaccharide components of the capsule. Adapted from Mandal et al. [96] under license https://creativecommons.org/licenses/by-nc/4.0/ (accessed on 22 January 2025).

The *C. neoformans* yeast cell is organized in distinct layers, each contributing to its functionality and pathogenicity (Figure 1). The innermost layer is the cell membrane that divides the cytoplasm from the extracellular milieu [76,77]. The cell membrane is surrounded by a cell wall consisting of glucans, chitin, and proteins, with distinct layers of polymerized melanin. Cell wall melanin protects the fungus from a variety of stresses [78,79]. β-glucans are crucial for maintaining cell viability and organizing the capsule [80,81]. Mannoproteins are highly immunogenic, stimulating T-cell responses, promoting cytokine production, and facilitating the adhesion of *C. neoformans* to host cells [82,83]. Chitin has been linked to ineffective Th2 immune responses, which can exacerbate disease progression [84]. Distal to the cell wall is the outermost layer, referred to as the polysaccharide capsule. This diffuse layer consists primarily of polymerized glucuronoxylomannan (GXM) and galactoxylomannan (GalXM) sugars that provide structural support, aid in adhesion to host tissues, suppress pro-inflammatory cytokines, block phagocytosis, and induce immune cell death [85,86,87,88,89,90]. Finally, the capsule is associated with antigenic variability [91,92,93,94,95]. Together, these structural components work in concert to enhance fungus survival and pathogenic potential.

#### 2.2.2. Extramembranous Virulence Factors: Polysaccharide Capsule and Cell Wall Melanin

The most well-understood *C. neoformans* virulence factor is the previously mentioned polysaccharide capsule [97]. This approximately 5 to 10 µm structure resides outside the cell wall and serves to shield the yeast from desiccation and phagocytic predation [98]. The *C. neoformans* capsule is a bona fide virulence factor, given that the absence of the capsule results in defective mammalian pathogenesis [99,100]. However, the capsule is not necessary for the yeast to live, as capsule-free *C. neoformans* has been shown to survive and replicate in vitro [101]. Early work revealed that challenge with capsular polysaccharides results in immunological unresponsiveness, mainly caused by an inhibition of antibody production [102,103]. Thus, a leading hypothesis is that the capsule shields the various pathogen-associated molecular patterns from innate immune recognition [104,105]. In addition, *C. neoformans* readily sheds its capsular polysaccharides, which possess immunomodulatory properties, including inhibiting neutrophil migration, altering cytokine production, modifying dendritic cell maturation, inducing apoptosis, and disrupting antigen presentation [89,106,107,108,109,110,111,112,113,114,115,116,117,118]. Finally, *C. neoformans* has the unique ability to alter the size and composition of its capsule in response to environmental or host-related conditions, a phenomenon known as capsule phase variation [119]. Environmental factors such as nutrient scarcity or stress and host-specific triggers like elevated CO_2_ levels, limited iron availability, and body temperature can drive these changes [119,120]. Capsule phase variation enables *C. neoformans* to thrive in the host environment and enhance its pathogenic potential [121]. For instance, a smaller capsule may strengthen initial tissue evasion or traversal of the blood–brain barrier (BBB), while a larger capsule provides robust protection against immune defenses during systemic infection [44,122]. This dynamic capsule regulation plays a central role in the organism’s virulence and its capacity to cause disease [97].

The second extramembranous *C. neoformans* virulence factor is the accumulation of cell wall melanin. Melanin is a dark pigment commonly found in animals and fungi that has demonstrated roles in many different infections and diseases [123]. Cryptococcal melanin is produced and polymerized within intracellular vesicles, known as melanosomes, before being transported to the cell wall [78,124]. Previous research suggests that strains of *C. neoformans* with higher melanin production are more capable of causing illness, whereas melanin-deficient mutant strains show a diminished ability to induce disease [125]. Melanin contributes to mammalian virulence by mediating resistance from free radicals, ionizing radiation, heat, and antifungal drugs [89,117,118,125,126,127,128,129]. Melanin has also been implicated in several key pathological processes, including facilitating fungal dissemination, altering host cytokine responses, and offering protection against macrophage-mediated defenses [130,131,132]. Interestingly, *C. neoformans* melanin is directly immunoreactive, as injection of melanin induces production of granulomas in the spleen, lungs, and liver, indicating that this pigment can induce pro-inflammatory responses [123]. These findings underscore the critical role of melanin in enhancing the pathogenic potential of *C. neoformans* and its ability to modulate host defenses, making it a key target for future therapeutic interventions. Together, the polysaccharide capsule and cell wall melanin are two primary structural components that serve as virulence mechanisms by disrupting the host immune response and providing protection against specific attacks orchestrated by the immune system [133].

#### 2.2.3. Phagocytosis Avoidance and Intracellular Pathogenesis

In addition to surface-associated virulence factors, *C. neoformans* also employs various strategies to evade host immune recognition and attack. These tactics enable the pathogen to persist within tissues and organs [133]. One such strategy was intimated above: prevention of phagocytosis. This phenotype is partially mediated by the fungal capsule but is aided by other antiphagocytic factors like antiphagocytic protein 1 (App1) [134]. However, instances where macrophages successfully engulf *C. neoformans* are not inevitably detrimental to the yeast [133,135]. An interesting characteristic of *C. neoformans* is that it is a facultative intracellular pathogen, meaning the yeast can resist phagolysosomal destruction and proliferate within myeloid cells [101,136]. This phenotype is linked to environmental survival of the yeast when subjected to engulfment by amoeba [137]. Fungal cells can continue replicating within both environmental and mammalian phagocytic cells [138]. In vivo, the intracellular macrophage–yeast interplay can lead to multiple different outcomes, including death of the yeast [101], *C. neoformans* proliferation and inflammatory lysis of the host cell [139], persistent co-existence [140], or a compromise where the fungus escapes from the host cell through non-lytic exocytosis called vomocytosis [141,142]. Vomocytosis is a unique mechanism by which *C. neoformans* escapes from macrophages while the macrophage remains alive [143,144]. This non-inflammatory process enables *C. neoformans* to persist within phagocytes without triggering strong inflammatory responses [145].

#### 2.2.4. Titan Cells

*C. neoformans* typically exists in its asexual (haploid) form as spherical budding yeasts but can also form short hyphae and spores during sexual reproduction [53,146]. In the absence of classical multicellular fungal differentiation phenotypes like molds and mushrooms, *C. neoformans* instead possesses the ability to retain its blastoconidial structure but grow into astoundingly large polyploid titan cells [44,147]. Approximately 20% of 5 to 10 μm *C. neoformans* yeast cells within the lungs differentiate into much larger ~100 μm titan cells [148,149]. Titan cells have been observed in human and animal models of cryptococcosis [148,149,150,151,152]. These giant cells evade macrophage phagocytosis, display a thickened cell wall, possess a denser capsule, and undergo significant alterations to the content and morphology of cytoplasmic organelles [43,45]. An important characteristic of these cells is that they are regulated via the protein kinase A (PKA) pathway, the same pathway that regulates the production of many other cryptococcal virulence factors, including the polysaccharide capsule and melanin [104,105,126,127,148,149,153,154].

While the existence of titan cells and genetically conserved signaling systems for cell differentiation is well established, their precise role during lung infection has not been elucidated. A leading hypothesis suggests that titan cells may directly facilitate the dissemination of typical-sized cryptococcal cells or that the normal-sized progeny of titan cells serves as a consistent source of replicative yeast during latent infection [43,44]. Numerous studies have consistently demonstrated that titan cells are a virulence factor for *C. neoformans* and are critical for the survival of the fungus [43,44,45,149,152]. However, further research is needed to clarify the exact role of these giant cells in *Cryptococcus* pathogenesis.

### 2.3. Cryptococcus Biofilm Formation and Contents

*C. neoformans* is commonly found in the environment. Consequently, cryptococcal cells have been exposed to various environmental insults and predation throughout the ecological history of the species [155,156,157]. This demanding environment has provided the necessary evolutionary pressure for the development of diverse and multi-situational defense mechanisms, specifically the ability to exist in protected biofilms [158]. Microorganisms often form biofilms as a strategy to endure challenging conditions, establish themselves in new habitats, and shield themselves from predation [159,160]. While cryptococcal biofilm formation likely evolved as a competitive advantage in stressful environmental settings, the ability to form biofilms also appears to be advantageous during human pathogenesis.

The discovery of medically relevant *C. neoformans* biofilms stemmed from a failure of a ventriculoatrial shunt that should have been draining cerebrospinal fluid into vascular circulation. Upon removal of the implanted medical device, ultrastructural studies revealed a biofilm consisting of yeast-like *Cryptococcus* [161]. Since this initial identification in 1980, a plethora of *C. neoformans* biofilms have been identified on various implanted medical devices, within the lungs, and in brain lesions [42,162,163,164,165,166]. These occurrences underscore the significance of biofilms in contributing to persistent *Cryptococcus* infections and highlight current and future treatment challenges. To address the rising incidence of *Cryptococcus* biofilms, scientists are increasingly focused on unraveling the complexities of these structures to better equip the medical community to combat them. In this section, we aim to highlight both the current knowledge and gaps in understanding surrounding *C. neoformans* biofilms.

#### 2.3.1. Structure and Maturation of Biofilms

**Figure 2:**
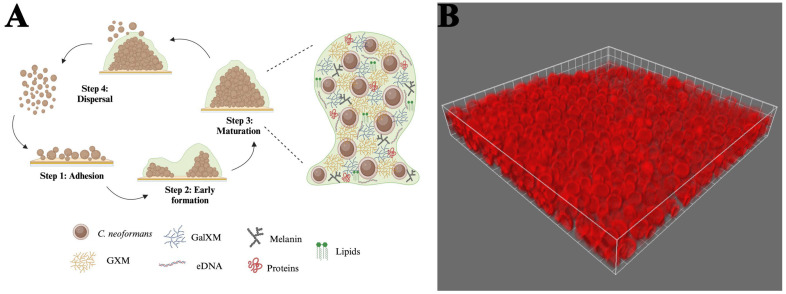
Stages of cryptococcal biofilm maturation and components. (**A**) (1) Adhesion: *C. neoformans* yeast cells adhere to a surface with the help of cell wall components. (2) Early formation: cells proliferate to form microcolonies; polysaccharide capsule production increases, providing a protective barrier; and extracellular matrix (ECM) deposition begins, comprising polysaccharides, proteins, DNA, and lipids. (3) Maturation: the biofilm structure becomes three-dimensional, with densely packed cells embedded in an extensive ECM. This ECM comprises key components, including GXM, GalXM, and melanin. (4) Dispersal: some cells revert to a planktonic state to colonize new areas. (**B**) A 63x immunofluorescent micrograph of *C. neoformans* 52D adhered to a glass coverslip and stained with Alexa Fluor 647-conjugated 18B7 antibody. Biofilm formation was performed according to Martinez and Casadevall with limited modifications [9].

*Cryptococcus* biofilms consist of yeast cells and somewhat elongated pseudohyphal cells embedded in an extensive ECM [167,168]. The characteristics of *C. neoformans* biofilm development were initially recorded using fungi that readily adhered to polystyrene microtiter plates. The authors described three coordinated phases of early biofilm development, including surface attachment, microcolony formation, and matrix production [11,26] (Figure 2). During the adhesion phase, metabolically active cryptococcal cells attach to a surface, forming a monolayer [11]. *C. neoformans* expresses several surface proteins, such as *Cfl1*, which contribute to its ability to adhere to surfaces [169,170]. The adhesion rate of the fungal cells can be enhanced by a conditioning layer composed of compounds released by the host [171]. This phenomenon has been observed in the brain, where cerebrospinal fluid surrounding a ventriculoatrial shunt contains increased amounts of cations. These positively charged ions may promote microbial adhesion to biomaterials, influencing the degree of fungal attachment [172]. During the intermediate stage, the yeasts divide, but the majority of the progeny remains associated with the microcolony. This process generates microcolonies with three-dimensional structures consisting of evenly dispersed yeast cells. The close proximity between the cells in these microcolonies assists in creating an environment conducive to establishing nutrient gradients, genetic exchange, and quorum sensing [11]. Throughout the final maturation stage, the architecture of these communities becomes more complex as the concentration of the ECM around the cells increases [9,11]. As the structures condense, they become more cohesive and adhesive [9,172,173]. Biofilm structural arrangement, along with the presence of flowing water channels, facilitates the exchange of nutrients and gases [8]. This mature structure offers fungal cells a protected environment, shielding them from environmental threats, immune responses, physical stress, and antimicrobial treatments [11].

An important factor involved in controlling biofilm development is the cell-to-cell communication known as quorum sensing, which affects biofilm formation by regulating cell growth, the release of GXM, and melanin synthesis [174]. *C. neoformans* employs different molecules that act as quorum sensing signals, including pantothenic acid (vitamin B5) and extracellular vesicles [174,175]. Alternatively, quorum quenching results in the disruption of quorum sensing pathways, preventing cells from coordinating biofilm formation and virulence [176,177,178]. Thus, developing solutions to target these pathways could weaken *C. neoformans* biofilms, making them more susceptible to antifungal drugs [179].

In addition to providing a physical barrier to antifungal agents, mature biofilms employ several maintenance and defense mechanisms. For example, *C. neoformans* can upregulate efflux pumps, which actively pump antifungal drugs out of the cells [180]. Furthermore, cells within the mature biofilm are generally less metabolically active, making them more resistant to drugs that target dividing cells [26]. Following biofilm maturation, some cells become planktonic and disperse into the surrounding environment, enabling them to colonize new areas [181]. Interestingly, these dispersed cells often exhibit transcriptional differences compared to their sessile counterparts within the biofilm. Specifically, genes involved in virulence, stress resistance, and metabolic pathways are upregulated in planktonic cells, priming them for survival and colonization in new niches [15,182]. Upon attaching to a new surface, the biofilm formation process reinitiates, and as the cells adapt to their surroundings, the biofilm structure evolves and reorganizes accordingly [183,184]. These stages highlight the dynamic and adaptive nature of *C. neoformans* biofilm development, emphasizing its role in persistence, environmental resilience, and potential for colonization of new surfaces.

In addition to transcriptional differences, *C. neoformans* biofilms harbor distinct populations of metabolically inert or dormant cells, sometimes referred to as persister cells [185]. These cells can survive extreme conditions, including nutrient deprivation and antifungal treatments, ensuring the survival of the biofilm even under adverse conditions [186,187,188]. These dormant cells may be critical for biofilm longevity, as biofilms can persist for weeks to months under suitable conditions, even in hostile environments such as those surrounding medical devices [50,189]. Moreover, the biofilm environment promotes increased opportunities for sexual reproduction and spore formation, particularly under nutrient-limiting conditions. The dense cellular proximity and genetic exchange within biofilms can enhance the likelihood of meiotic events, resulting in the production of stress-resistant spores capable of dispersal and colonization [190].

#### 2.3.2. In Vivo *C. neoformans* Biofilms

*C. neoformans* primarily forms biofilms on abiotic surfaces like medical devices. Demonstrated biofilm substrates include prosthetic cardiac valves, prosthetic dialysis fistulas, and prosthetic hip joints [162,164,165]. In addition, it is believed that *C. neoformans* may produce biofilm-like clusters within the lungs and brain [163,191]. Cranial *C. neoformans* infections include biofilm-like structures known as cryptococcomas [192,193,194]. The development of these structures occurs as a result of uncontrolled infection and plays a vital role in fungal neurotropism [42]. The fungal communities within cryptococcomas are characterized by substantial amounts of capsular polysaccharides surrounding yeast cells and can appear as cystic lesions in the brain [42,195]. Further research into biofilm formation on lung tissue and the role of cryptococcomas in neurotropism may provide valuable insights into the pathogenesis of *C. neoformans* and its impact on both systemic and localized infections.

#### 2.3.3. Extracellular Polysaccharides in Cryptococcal Biofilm Development

Many of the factors that contribute to *C. neoformans* pathogenesis also aid in biofilm formation. A major virulence factor discussed earlier that supports biofilm development is the polysaccharide capsule. Substantial amounts of capsular polysaccharide are shed during biofilm formation and become a main component of the extracellular matrix (ECM). This release creates the three-dimensional framework of the ECM that encases and protects the embedded cells to ensure preservation and stability [9,172]. This is evidenced by the finding that the acapsular cap59 mutant strain does not form biofilms, while the encapsulated wild-type and complement strains exhibited similar biofilm-forming abilities. Since the primary component of the *C. neoformans* capsule is a key element of the extracellular biofilm matrix, the authors concluded that the mutant strain’s failure to form biofilms is due to its inability to shed polysaccharides [9].

The cryptococcal ECM is composed primarily of the capsular polysaccharides (GXM and GalXM) but also contains various proteins, including laccases and phospholipases, extracellular DNA (eDNA), lipids, and melanin. Other molecules, including metabolites and ions, can also be sequestered within the ECM [196]. Glycoproteins like laccase, mannoproteins, and phospholipase B1 (PLB1) play vital roles in biofilm formation, structural integrity, antifungal resistance, and interactions with the host immune system. Laccase contributes to melanin production, mannoproteins mediate adhesion to surfaces and trigger immune modulation, and PLB1 facilitates biofilm integrity and virulence [48,82,83,197,198,199,200]. eDNA is also a critical component of the fungal ECM [201,202,203], and its essentiality was established when Whitchurch et al. demonstrated that enzymatic degradation of eDNA decreased biofilm biomass, increased susceptibility to antifungal treatments, and resulted in structural disintegration of the biofilm matrix [204]. Since then, eDNA has been recognized in the importance of biofilm formation, structural stability, and adhesion and signaling between cells [205]. While there is still significant debate as to whether eDNA is actively secreted or derived from lysed cells, this extracellular nucleic acid contributes to the resilience of *C. neoformans* in both environmental and host settings [202,206,207]. Together, capsular polysaccharides, proteins, and eDNA are the major components of the *C. neoformans* biofilm.

#### 2.3.4. Connection Between Intracellular Survival of *C. neoformans* in Macrophages and Biofilm Development

Many of the virulence factors associated with *C. neoformans* survival within macrophages likely also contribute to biofilm formation. First, mobile mononuclear host cells likely act as a vehicle for *C. neoformans* dissemination via a mechanism named the “Trojan horse” [208]. Here, intracellular yeasts can exit macrophages via lytic or non-lytic exocytosis, and these released cells are primed to establish biofilms at new sites [139,141,142]. Second, intracellular challenges like limited nutrients and oxidative stress induce *C. neoformans* to adapt by initiating biofilm formation [27,42]. Finally, intracellular *C. neoformans* produces capsule polysaccharides, enabling those yeasts that escape the phagocyte to immediately contribute to biofilm formation [121,209]. These released cells can aggregate and form biofilms on host tissues or medical devices, using quorum-sensing signals to coordinate biofilm development [11]. Taken together, these factors suggest that intra-macrophage survival modulates the yeast to elaborate many of the virulence mechanisms that result in biofilm initiation. This dichotomy underscores the ability of *C. neoformans* to transition between different survival strategies, contributing to its pathogenicity, persistence in the host, and resistance to treatment. Understanding this connection offers potential targets for disrupting these processes to improve therapeutic outcomes.

Interestingly, the binding of antibodies to the outer layers of *C. neoformans* can contribute to biofilm formation by agglutinating cryptococcal cells into biofilm-like microcolonies [210]. While this cell clumping is notable, anti-capsule antibodies are not the primary contributors to biofilm formation and, therefore, are not particularly effective at inducing it. Microcolonies play a crucial role as foundational units of biofilms, facilitating the spread of *C. neoformans* to new environments. As discussed in a previous review paper, the fungus may establish biofilms within tissues in these areas, enter a dormant state, and evade clearance by macrophages, antimicrobial therapies, or immune cell molecules [42]. This phenomenon is particularly pronounced in the brains of individuals with cryptococcosis, where cryptococcomas are formed and play a vital role in the pathogen’s neurotropic success [42,211].

#### 2.3.5. Involvement of Titan Cells in the Biofilm Generation

As integral parts of lung biofilms, the thickened cell wall and capsule of titan cells aid in restricting host immune responses and contribute to overall structural integrity [45]. Additionally, studies suggest that these large cells can confer protective benefits not only to themselves but also to normal-sized cryptococcal cells [45]. This is supported by the fact that *C. neoformans* biofilm formation protects densely-packed cryptococci from antimicrobial damage and macrophage phagocytosis in tissues, enhancing fungal resistance, quorum sensing, and survival [9]. Therefore, titan cells may contribute to the initial stages of biofilm formation by adhering to surfaces and facilitating the aggregation of other cryptococcal cells.

In *C. neoformans*, titan cell formation is triggered by the same environmental conditions—such as high CO_2_, low oxygen, and nutrient limitation—and genetic factors, including the cAMP signaling pathway, that drive biofilm development [45,212,213,214,215,216,217,218]. These processes serve as adaptive responses to stress, which enhance fungal survival in hostile environments. Titan cells, being larger and more resistant to immune responses, contribute to biofilm formation by enriching the extracellular matrix with polysaccharides [42]. This overlap in conditions and pathways illustrates a coordinated survival strategy, allowing the fungus to persist in the host and establish chronic infections.

### 2.4. C. neoformans Biofilms in Human Infections

#### 2.4.1. Contribution of *C. neoformans* Biofilms to Implanted Medical Device Infections

*C. neoformans* forms biofilms on a wide range of synthetic materials, including catheters, prosthetic valves, and other indwelling medical devices. These biofilms are facilitated by the ability of the organism to produce a polysaccharide capsule and secrete exopolysaccharides that contribute to the ECM [15,28,219]. Biofilms provide an ideal niche for *C. neoformans* to evade antifungal treatments through ECM-mediated drug sequestration, metabolic heterogeneity, and reduced drug penetration [220,221]. Infections involving *C. neoformans* biofilms on medical devices are difficult to eradicate and often require removal of the infected device [221,222,223]. These infections are associated with high morbidity and, in some cases, mortality [15,220,221]. This increasing prevalence of biofilm formation on indwelling medical devices demonstrates the urgent need for antifungal strategies targeting biofilm-associated infections.

#### 2.4.2. Contribution of Biofilms to *C. neoformans* Brain Infections

**Figure 3:**
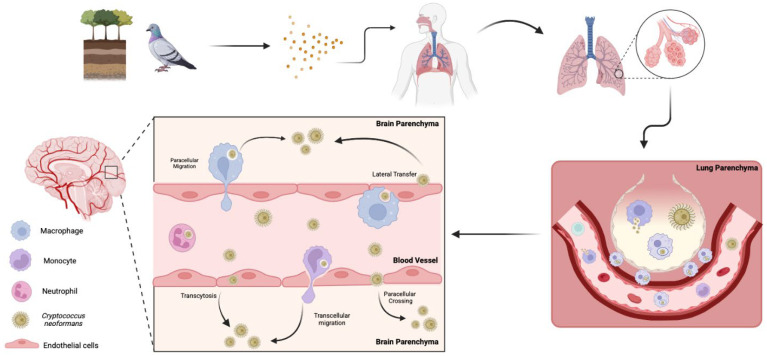
Pathways of *C. neoformans* invasion into the brain. Spores from soil or bird excreta are inhaled into the lungs, the primary site of infection. If the infection is not cleared, *C. neoformans* yeasts may transverse the lungs and enter the bloodstream. This occurs either through direct fungal migration into the blood or via mobile phagocytes that engulf the fungus and transport it across barriers. Once in circulation, the fungus disseminates throughout the body, with a predilection for the central nervous system. *C. neoformans* crosses the blood–brain barrier via multiple mechanisms, including transcytosis, paracellular crossing, and lateral transfer. Additionally, the fungus employs “Trojan horse” strategies in which phagocytes carry fungal cells crossing the blood–brain barrier via transcellular and paracellular migration.

Infiltration of *C. neoformans* into the central nervous system (CNS) can result in fatal meningoencephalitis. Thus, it is imperative to understand the factors that contribute to the mechanisms by which this fungus disrupts the blood–brain barrier (BBB). There are three main theories for the mechanism(s) of cryptococcal brain invasion; the fungus can cross transcellularly through endothelial cells [224], paracellularly between endothelial cells [224], or through lateral transfer, where phagocytes in the bloodstream release fungal cells at the BBB, allowing them to cross. It also has the unique capability to employ what is known as the “Trojan horse” mechanism, where infected macrophages carry the fungus across the BBB [208,225] (Figure 3). From the fungal perspective, the lung, BBB, and brain are all different environments, as exemplified by the oxygen-rich lung environment compared to the low-oxygen brain parenchyma. Cryptococcal cells may prefer the oxygen-rich environment, as the fungi tend to occupy areas near capillary-rich endothelial cells within the brain [226]. However, the oxygen-depleted environment within the brain likely induces similar signaling events to those occurring in biofilm formation in other host environments, ultimately leading to morphological adaptations that enhance persistence [224].

Within the CNS, cryptococcal biofilm-like structures may form along endothelial surfaces or in association with immune cells, contributing to persistent infection and recurrent disease [227]. Additionally, these biofilm structures can serve as a reservoir for episodic dispersal of fungal cells, potentially allowing for re-infection of the brain parenchyma [228]. The hypoxic and nutrient-limited environment of the CNS may further promote the transition to biofilm-associated states, as observed in other pathogenic fungi [226,229]. These diverse mechanisms, combined with the critical role of biofilm formation in protecting the fungus and promoting persistent infection, highlight the adaptability of *C. neoformans* and underscore the challenges in effectively treating cryptococcal infections in the CNS. Ultimately, the intricate interplay between biofilm formation, immune evasion, and the ability to disseminate undetected presents significant obstacles in managing cryptococcosis.

## 3. Other Pathogenic Fungi That Form Biofilms

### 3.1. Candida *spp.*

*Candida albicans* is undoubtedly the most conspicuous example of a pathogenic fungus that forms biofilms during infection. As a natural component of the human microbiota, *C. albicans* is typically found in the gastrointestinal tract, female reproductive tract, oral cavity, and skin [31,230]. However, when the host/fungus equilibrium is disrupted by factors such as environmental changes (e.g., pH or nutrient shifts), antibiotic use, or immune system alterations (from infections or immunosuppressive treatments), the fungus can rapidly proliferate, leading to candidiasis [231]. The symptoms of candidiasis range from mild conditions like oral skin rashes and genital infections to severe cases with fever, chills, and, potentially, organ failure [16,232]. Candidiasis often leads to an infection known as thrush, characterized by a white biofilm on the tongue, throat, or surrounding areas of the mouth [233]. Extensive research on oral thrush has greatly advanced the general understanding of biofilm resilience and the development of therapeutic strategies for biofilm-related diseases [234].

*Candida* species can also form biofilms in other circumstances, particularly in hospital settings [21]. *C. albicans* and other closely related species are the predominant fungi isolated from infected medical devices and are among the leading causes of bloodstream infections [235]. Among patients with invasive candidiasis, mortality rates remain high, with approximately 40% succumbing to the infection despite antifungal treatment [236]. Thus, secondary candidiasis has become a critical focus in hospital settings, as *C. albicans* biofilm formation accounts for roughly 15% of all sepsis cases and 40% of bloodstream infections [235]. Quorum sensing plays a crucial role in *C. albicans* by regulating its transition between yeast and hyphal morphologies, a key factor in its virulence and biofilm formation [237]. *Candida* biofilms are notoriously difficult to treat, often requiring device removal and high doses of antifungal drugs, which carry risks such as organ damage [16,222,223]. The resilience of *Candida* biofilms highlights the urgent need for new therapeutic strategies to combat these persistent infections, especially in critically ill and immunocompromised patients.

### 3.2. Aspergillus *spp.*

*Aspergillus* species such as *A. fumigatus*, *A. flavus*, *A. niger*, and *A. terreus* are known to form harmful biofilms, which contribute to various diseases in humans, birds, and mammals [17]. *A. fumigatus* is among the most common airborne fungal pathogens in humans and is primarily associated with pulmonary disease [5]. However, *A. fumigatus* infections can result in various conditions ranging from rhinitis to fatal invasive aspergillosis in immunocompromised patients [238,239,240]. Aflatoxin-producing *A. flavus* can cause severe respiratory issues and allergic reactions [241,242]. The black mold *A. niger* can lead to aspergillosis in immunocompromised individuals [243]. Finally, *A. terreus* is notable for its growing prevalence and resistance to certain antifungal agents [244]. The incidence of aspergillosis has risen, with some strains showing reduced susceptibility to antifungal treatments, likely due to biofilm-associated drug resistance [242,245,246,247].

The formation of biofilm by *Aspergillus* species is a key factor in their virulence and plays a significant role in antifungal resistance [248,249]. *Aspergillus* biofilms differ somewhat from classical biofilms due to the presence of differentiated hyphae, but these communities share many characteristics of all biofilms, including ECM production, surface adherence, and enhanced antifungal resistance [17]. Therefore, the term “*Aspergillus* biofilm” has become widely accepted because these communities share similar clinical and industrial challenges with classical biofilms [250]. Biofilms provide a sheltered niche for the fungi, protecting them from immune cells, environmental predators, and shear forces [251]. Despite their importance in *Aspergillus* infections, the biofilm trait remains underexplored, with most research focusing on the conidial form of the organism [17]. A deeper understanding of *Aspergillus* biofilms is crucial to address their ability to evade immune responses and resist antifungal therapies.

### 3.3. Malassezia *spp.*

Many different lipophilic yeasts in the genus *Malassezia* can form biofilms. *Malassezia* spp. are commonly associated with the skin of humans and various mammals, where the fungi live in close association with fat-producing sebaceous glands [158,252,253]. Normally, *Malassezia* spp. are considered part of the normal skin microbiota, as most interactions are commensal. However, changes to this host/fungus equilibrium can lead to minor disorders like dandruff, seborrheic dermatitis, otitis externa, and dermatitis [254,255,256] or more severe infections like fungemia, catheter infections, and severe nosocomial infections [18,253,257]. This change from commensal to opportunistic pathogen correlates strongly with the formation of biofilms with nearly 2000 times higher resistance to antimicrobial therapeutics [257,258,259].

*Malassezia* biofilms are commonly found on medical devices, particularly catheters, and are associated with serious infections in immunocompromised patients [18,253]. The catheter-related disease is classified as a nosocomial infection, affecting many intensive care unit patients, and is often associated with high mortality rates [257]. It is important to note that a significant proportion of nosocomial infections are due to biofilm formation [18,257]. Given the rising incidence of biofilm-associated infections and the unique challenges posed by *Malassezia* species, further research is essential to better understand their pathogenic mechanisms and improve treatment strategies for affected patients.

### 3.4. Trichosporon *spp.*

*Trichosporon* spp. are comparatively less commonly pathogenic biofilm-forming fungi. While long known to be a member of the skin microbiota, *Trichosporon* fungi have emerged as a significant public health concern in immunosuppressed patients [260,261]. In rare circumstances, *Trichosporon* can cause a life-threatening condition known as disseminated trichosporonosis, where the fungus spreads throughout the body, damaging multiple organs and systems. It most commonly affects those with compromised immune systems but can also affect healthy people under certain conditions [23,260]. It was found that most of the reported cases of *T. asahii* infections correlated with the presence of indwelling devices such as intravenous or urinary catheters [262], endoscopic forceps [263], and arteriovenous grafts [264]. These findings corroborate the notion that prosthetic devices can act as an ideal substance for the adhesion and growth of biofilms.

Similar to the other fungal species mentioned thus far, *Trichosporon* biofilms are often associated with persistent infection and high mortality [23]. Several *Trichosporon* species form biofilms [25], the most common one being *T. asahii.* Biofilms comprising this fungus have been seen to form on various medical devices [265]. *T. ashaii* and *T. inkin* both form biofilms that are resistant to classic antifungals [19,266]. A unique characteristic of the biofilms formed by these species is their ability to form persister cells [267]. These cells can enter a dormant state or express certain genes for inactivation, enabling them to exhibit antimicrobial tolerance [268]. *Trichosporon*-specific biofilms result in the same consequences seen in other fungal biofilms, marked by enhanced resistance to antimicrobial agents and protection against host immune defenses.

### 3.5. Fusarium *spp.*

Other fungi that can form biofilms are members of the *Fusarium* genus, including *F. oxysporum* and *F. solani* [20]. *F. solani* is one of the most commonly isolated filamentous fungi from soil and plant debris [269]. While these species are primarily saprophytic, they also serve as host-specific pathogens for many agricultural plants and have been sporadically associated with severe and invasive mycoses in immunocompromised human patients [269,270,271,272,273]. *F. oxysporum* is distinguished by its capacity to form biofilms on various unique surfaces, including contact lenses and water systems [274,275]. However, it is most notable for its formation of biofilms on human nails, leading to an infection known as onychomycosis [276].

Instances of *Fusarium* infections have increased in recent decades. This rise may be attributed to several factors, such as a growing population of immunocompromised patients [272], rising medical tourism [277,278], climate change [279], and resistance to antifungal treatments [280]. Interestingly, the recent 2023 outbreak of *F. solani* revealed a trend that diverges from previous observations, whereby immunocompetent patients developed meningitis [277]. This outbreak differed significantly from past occurrences involving *Fusarium*, mainly because this fungus usually targets immunocompromised individuals and because cases of fusariosis in the CNS are seldom documented [278]. Due to their ability to form biofilms, *Fusarium* species are resistant to current antifungal interventions, making infections all the more difficult to treat [280]. As the complexities of *Fusarium* infections continue to evolve, advancing our understanding of their biofilm formation is essential for developing more effective diagnostic and therapeutic approaches.

### 3.6. Coccidioides *spp.*

*Coccidioides* spp. also possess the ability to form disease-causing biofilms. *C. immitis* and *C. posadasii* both cause a lung infection called valley fever and pulmonary coccidioidomycosis [281,282]. *Coccidioides* is endemic to arid and semi-arid regions of the southwestern United States, Mexico, Central America, and South America [283]. The majority of coccidioidomycosis cases resolve without significant complications, but rare chronic infections can persist for a lifetime, occasionally causing additional health issues [284]. Coccidioidomycosis most commonly presents as an upper respiratory infection; however, it may also lead to disseminated disease, which has been proven to be treatment-resistant and thus potentially fatal [285]. As with the previously discussed fungal infections, this illness can infect immunocompetent individuals but is considerably more common in immunocompromised patients [283]. Due to its similar presentation of pneumonia, outbreaks of coccidioidomycosis are often misdiagnosed and are, therefore, a common cause of community-acquired pneumonia [283].

*Coccidioides* spp. can adhere to and form biofilms on medical devices [22]. A case study documented recurrent coccidioidal meningitis caused by a fungal biofilm on the tip of a ventriculoperitoneal shunt tubing despite the patient receiving an adequate dosage of fluconazole, highlighting the role of biofilms in treatment failure [22]. The authors described how the biofilm structure likely shielded the fungal cells from the antifungal drug and host immune response, complicating treatment outcomes. Biofilm formation in *Coccidioides* shares similarities with other pathogenic fungi, such as their ability to attach to biomaterials and resist antifungal treatments [286]. Due to the specific region that this fungus resides in, along with the fact that it is so challenging to diagnose, there is limited information available regarding *Coccidioides* biofilms [221]. Given the challenges in diagnosis and treatment associated with *Coccidioides* infections, further research into its biofilm formation and pathogenic mechanisms is crucial for improving management strategies and patient outcomes.

## 4. Current and Future Treatment Strategies for Fungal Infections

### 4.1. Current Fungal Infection Treatment Tools

There are four primary classes of antifungal drugs available to treat fungal infections [287] (Table 1). Polyenes, such as amphotericin B (AmB), target ergosterol in fungal membranes, causing a leakage of cellular components [288,289,290,291]. While AmB remains the principal drug of choice for systemic infections, a major limiting factor in its efficacy is its negative side effects. The extensive use of AmB is correlated with increased nephrotoxicity, though its liposomal formulation can reduce adverse reactions [292,293]. Azoles inhibit lanosterol 14-alpha-demethylase, disrupting ergosterol biosynthesis, and are commonly used for cutaneous *C. albicans* infections due to their accessibility and low dermal toxicity [294,295]. Although less toxic than AmB, azoles are associated with hepatotoxicity by affecting key enzymes involved in regulating liver activity [296]. Echinocandins block β-1,3-D-glucan synthase, an enzyme necessary for the synthesis of β-1,3-D-glucan, a major constituent of the fungal cell wall. While effective against *Candida* and *Aspergillus*, they are not effective against *C. neoformans* [297,298,299]. Despite having a more favorable tolerability profile compared to polyenes and azoles, echinocandins are not without drawbacks. Studies have linked their use to potential cardiotoxicity [300]. Furthermore, the high cost of echinocandins and the absence of oral formulations significantly limit their widespread application in clinical practice [301]. Allylamines, exemplified by terbinafine, disrupt ergosterol biosynthesis by targeting the enzyme squalene epoxidase [302,303]. This class of antifungals boasts a favorable safety profile and is primarily employed in treating superficial dermatophyte infections. However, their effectiveness against systemic *Candida* and *Cryptococcus* infections remains limited, constraining their use in such cases [304,305]. While conventional antifungal drugs are effective against planktonic fungi, only echinocandins and liposomal AmB demonstrate efficacy in combating biofilm-associated infections and may be used in combination with other therapies to elicit a greater effect [306]. However, even these treatments may result in suboptimal outcomes, underscoring the urgent need for more advanced therapeutic strategies to address invasive fungal infections [306,307,308].

As it pertains to *Cryptococcus* infections, the WHO has recently revised its recommended induction therapy for cryptococcal meningitis to include a single high dose (10 mg/kg) of liposomal AmB, administered alongside 14 days of flucytosine (100 mg/kg per day, divided into four doses) and fluconazole (1200 mg daily for adults, 12 mg/kg per day for children and adolescents, up to a maximum of 800 mg daily) [73]. Unfortunately, certain elements of this treatment remain inaccessible in some regions, particularly in areas with high disease prevalence, such as sub-Saharan Africa [309]. The WHO has published alternative guidelines for when certain medications are not available [73]. Its guidelines for the consolidation phase include fluconazole (800 mg daily for adults, 6–12 mg/kg per day for children and adolescents, up to a maximum of 800 mg daily) for eight weeks following the induction phase [73]. For the maintenance therapy phase of treatment, they recommend fluconazole (200 mg daily for adults, 6 mg/kg per day for adolescents and children) until immune reconstitution (CD4 > 200 cells/mm^3^) and suppression of viral loads on ART [73].

### 4.2. Investigational and Proposed Fungal Biofilm Therapeutics

Fungal biofilms are an emerging public health threat, prompting significant efforts to identify effective treatments for biofilm-associated infections [310]. The current challenges can be summarized in six major themes: (1) Well-structured biofilms cause drastic changes in the genetic and metabolic states of cells, with yeast cells deep within the biofilm being less metabolically active and thus more resistant to antifungal treatment [11,50]. (2) Biofilms on medical implants are inherently resistant to phagocytosis and immune clearance [311]. (3) *C. neoformans* can reproduce both sexually and asexually, leading to genetic, morphological, and phenotypic variations that enhance its adaptability [146]. (4) Biofilms adhere strongly to surfaces, making them resistant to chemical or physical inactivation [312,313]. (5) Fungal biofilms serve as persistent sources of infection, shedding yeast cells that can spread to other areas within the host [26,314]. (6) Rapid disruption of biofilms may release an excess of immunostimulatory molecules, potentially triggering autoimmune diseases [107,315]. These characteristics of fungal biofilms present an immediate and noteworthy challenge for physicians and scientists alike. In response, researchers have begun exploring various strategies to tackle the challenge of fungal biofilms, with the primary goal of preventing biofilm formation, as mature biofilms are notably more resistant to treatment [316].

#### 4.2.1. Antifungal Lock Therapy

A promising development is the use of catheter lock therapy. The extensive use of indwelling catheters, particularly intravenous catheters, has greatly contributed to the increase in bloodstream infections resulting from both bacterial and fungal biofilms [317]. Infections resulting from biofilm formation on catheters usually require removal of the tubing and systemic antifungal therapy to treat the disease effectively [318]. However, an alternative approach, known as antimicrobial lock therapy (ALT), has gained traction in recent years. The premise behind ALT is that it utilizes extended delivery of a solution that contains high concentrations of antimicrobial or antiseptic agents. The mixture is then introduced into an infected intravascular catheter, often alongside systemic antifungal therapy, to disinfect the catheter [318]. Previous reviews have outlined some of the most promising antifungal lock strategies involving the use of conventional antifungal drugs like AmB and echinocandins, antibiotics with antifungal properties such as doxycycline, antimicrobial peptides (AMPs), and antiseptics, particularly ethanol [318,319,320]. ALT has shown success in both in vitro and in vivo studies with *Candida* biofilms [321,322,323]; however, clinical studies for *C. albicans*, specifically, remain limited, with even less research available for *C. neoformans* biofilms. Therefore, further research on ALT as a means of treating biofilm-associated infections must be conducted to ensure the safe implementation of this treatment into clinical practice.

#### 4.2.2. Nanoformulations

A novel approach involving nanocomposites has been emerging as a promising treatment for fungal biofilms due to their ability to disrupt biofilm structure, enhance drug delivery, and exhibit antimicrobial properties [324]. One of the key advantages of nanomedicine is the use of nanocomposite-coated or tethered biomaterials, which can prevent fungal attachment and colonization, thereby reducing infection risks. Nanotherapeutics involve newly developed antifungal agents and enhance the effectiveness of existing drugs by improving their bioavailability, targeted delivery, solubility, and stability [325,326]. Notably, recent work has shown that newly engineered nanocomposites can directly target fungal biofilms, a capability that many conventional antifungal therapies lack due to their inability to penetrate these protective structures [327]. Nanocomposites are constructed by embedding nanomaterials into a host material to create an intricate three-dimensional structure [328]. By taking advantage of the protective biofilm structure, nanocomposites encapsulate antifungal drugs, bypass biofilm defenses, and facilitate drug delivery to the cell surface or within the cell, thereby enhancing the effectiveness of traditional treatments [329]. Nanocomposite therapies are advantageous, as they are structurally diverse and can be administered through nearly all routes. However, implementing this in clinical practice is challenging due to its inherent instability and the complexities involved in achieving precise, large-scale production [324]. Furthermore, even minor alterations in their spatial configuration can significantly alter their safety and effectiveness, often resulting in clinical trial failure [330]. While nanomedicine has shown potential in commercialization, its use for wide-scale implementation is still quite limited. The primary reason for this is the absence of clear guidelines and regulations for producers, medical personnel, and international public health agencies [331]. Thus, while nanocomposites show great promise in overcoming the challenges posed by fungal biofilms, their clinical implementation faces significant hurdles, including stability issues and the lack of comprehensive regulatory frameworks. Addressing these obstacles will be crucial for the successful commercialization and integration of nanomedicine into standard antifungal treatments. 

#### 4.2.3. Modified Surfaces and Antimicrobial Coatings

Various biomaterials commonly utilized in medical devices have been found to facilitate fungal attachment, proliferation, and biofilm production. [316,332]. These materials include silicone, polyurethane, polyethylene, polypropylene, polymethylmethacrylate, titanium, titanium alloys, stainless steel, and polytetrafluoroethylene [333,334]. Over time, scientists have identified three major factors influencing biofilm formation: (1) surface roughness, (2) hydrophobicity, and (3) protein adsorption [333]. Rough surfaces offer increased surface area for microbial attachment, hydrophobic materials attract fungi due to their affinity for similar properties, and many implants adsorb proteins from bodily fluids, creating a conditioning layer that promotes fungal adhesion [335]. Although fewer studies focus exclusively on antifungal biomaterials compared to antibacterial ones, some significant discoveries have been made in this arena. Specifically, surface coatings that incorporate antifungal agents, such as cationic compounds, low-molecular-weight antiseptics, antimicrobial peptides, and polyenes, bind to the surface [336]. When these agents are covalently bound to the biomaterial surface, they are neither released nor damaged and, in theory, should persist at high concentrations to continuously kill or inhibit microorganisms upon contact [336]. This concept has also been explored with bacterial biofilms. Numerous studies have proposed using natural or modified polysaccharide polymers as surface treatments to inhibit biofilm formation [337,338,339]. The use of modified surfaces and antimicrobial coatings represents a promising approach to preventing fungal biofilm formation on medical implants, and ongoing research into antifungal surface treatments continues to uncover innovative strategies for enhancing the effectiveness of these materials.

#### 4.2.4. Natural Remedies

Natural products have also been explored as possible treatment options for fungal biofilms. The development of microtiter plate-based models of fungal biofilm formation and antifungal susceptibility testing has led to an increase in research investigating the antifungal properties of natural products on biofilms. One study showed that a plant-derived decapeptide, known as OSIP108, interferes with *C. albicans* biofilm formation without affecting the viability or growth of the individual cells [340]. While research has predominantly focused on *C. albicans*, recent studies have highlighted several promising organic strategies for combating *Cryptococcus* biofilms. Notably, ethanolic extract propolis has been shown to effectively reduce biofilm formation by *C. neoformans* [200]. Additionally, researchers have identified natural compounds derived from freshwater mussels that disrupt key factors of fungal virulence. These compounds not only reduce polysaccharide capsule production and inhibit biofilm formation but also enhance the fluconazole susceptibility of *C. neoformans* in the presence of macrophages [341]. Another noteworthy area of interest is chitosan, a polymer derived from chitin found in crustacean exoskeletons. Chitosan has demonstrated promising results in disrupting biofilms formed by both *C. albicans* and *C. neoformans*, both in vitro and in vivo [173,342]. Interestingly, this compound also exhibits antibiotic properties and has been observed in layered formations with other polysaccharides that create coatings that inhibit binding and are bactericidal [337]. Moreover, certain natural products have the potential to enhance the efficacy of conventional antifungal drugs. For instance, a study revealed that shearinines, a group of structurally complex molecules produced by fungi in the *Penicillium* genus, significantly enhanced the activity of AmB against *C. albicans* biofilms in clinical isolates [343]. This multifaceted approach highlights natural remedies as promising tools for combating fungal biofilms, with studies showing their potential to inhibit biofilm formation, enhance antifungal efficacy, and reduce virulence. Further exploration into natural products is essential for developing complementary or alternative treatments.

#### 4.2.5. Advances in Biofilm Detection and Doctor Education

Implant-associated infections pose a significant problem for the healthcare industry, as they are exacerbated by the formation of microbial biofilms and are often difficult to diagnose [344,345]. Advancements in detecting fungal biofilms on implants have revolutionized diagnostic capabilities [15]. The agar encasement culturing method combined with the candle dip method is a notable innovation, allowing for the visualization and precise localization of biofilm formation on endoprostheses [346]. This technique identifies susceptible implant areas and facilitates further analysis of isolated colonies. High-resolution scanning electron microscopy has become a leading tool for detailed microscopic analysis, successfully visualizing bacterial biofilms on infected cochlear implants and offering insights into biofilm structure and composition [347,348]. Additionally, sensor-based approaches are emerging as accurate and user-friendly methods for detecting and monitoring biofilms at implant sites. These portable technologies are particularly valuable in resource-limited settings. Machine learning and AI-driven deep learning models also hold great potential for identifying polymicrobial biofilms, surpassing the 50% accuracy of human specialists [349]. These innovations provide efficient alternatives to traditional, time-intensive biochemical procedures, significantly enhancing biofilm management in clinical environments worldwide.

Efforts to educate healthcare providers in underserved areas about fungal biofilm infections are increasingly recognized as vital, given the impact of social determinants of health on fungal disease prevalence. While specific initiatives remain limited, broader frameworks offer opportunities for integration. For instance, the WHO has advocated for incorporating fungal diseases and priority pathogens into medical and public health curricula [350]. Such initiatives could be expanded to address fungal biofilm infections, equipping doctors in resource-constrained settings with the knowledge needed to manage these challenging infections effectively.

## 5. Concluding Remarks

Fungal biofilms have emerged as a significant concern in both clinical and environmental settings, as these structured communities exhibit increased resistance to antifungal therapies and the immune system. These biofilms are formed by various fungal species, such as *Cryptococcus*, *Candida*, and *Aspergillus*, and contribute to chronic infections that are difficult to treat. The biofilm matrix provides physical protection, limits drug penetration, and promotes genetic exchange, which can further drive antifungal resistance.

Fungal species that can form biofilms complicate treatment strategies, as established biofilms often require higher doses of antifungal drugs and are more resistant to the host immune response. This is particularly relevant in healthcare-associated infections, where biofilms can lead to severe complications. The ability of fungal biofilms to persist on a diverse range of surfaces, such as medical devices, tissues, and environmental areas, poses a significant challenge for researchers. This lack of effective treatments to fully eradicate biofilms highlights the need for novel therapeutic approaches, such as targeting biofilm-specific pathways or utilizing combination therapies.

Cryptococcal biofilms are especially important because they are associated with increased resistance to antifungal treatments, including commonly used drugs like azoles and polyenes, leading to poor therapeutic outcomes. This resistance, combined with a global increase in cases of cryptococcosis and the high mortality rate of cryptococcal meningoencephalitis, underscores the urgency of developing effective treatments that can target these resilient biofilms.

It is important to note that there is still much that is unknown regarding fungal biofilms and their interaction with the host immune system. Future research must focus on better understanding the molecular mechanisms underlying biofilm formation, as well as identifying novel therapeutic strategies capable of disrupting or preventing biofilm development. The growing prevalence of fungal diseases in both healthy and immunocompromised individuals, particularly as a result of biofilm formation, has resulted in a surge in research investigating these infections. With ongoing advancements, there is promise that more effective treatments can be developed to reduce the morbidity and mortality associated with fungal biofilm infections.

## Figures and Tables

**Table 1:** Table summarizing the characteristics of each fungal group.

Fungal Species	Natural Habitat	Infections Caused	Common Biofilm Locations	Biofilm-Associated Complications	Biofilm Resistance Characteristics	Current Treatments
*Cryptococcus neoformans*	Soil (often pigeon droppings)	Cryptococcosis, cryptococcal meningoencephalitis	Ventriculoatrial shunts, heart valves, CNS tissue	Persistent CNS infections, biofilms on medical devices, brain cryptococcomas	Enhanced survival in macrophages, immune evasion in brain, extreme resistance in CNS biofilm structures	Primary treatment involves AmB with flucytosine for meningitis, maintenance therapy with fluconazole to prevent recurrence. Device removal necessary for biofilm infections
*Candida* spp.	Human microbiota (GI tract, skin, oral, reproductive tracts)	Candidiasis: thrush, genital infections, invasive candidiasis	Medical devices, GI tract, female reproductive tract, oral cavity, skin	Biofilm formation on medical devices leading to sepsis, bloodstream infections	High resistance to antifungals, challenging treatment due to recurrent infections	Removal of infected medical devices, high-dose antifungal therapy (azoles, echinocandins, AmB)
*Aspergillus* spp.	Soil, compost, airborne	Aspergillosis: sinusitis, rhinitis, invasive aspergillosis (IA), fungal keratitis, otomycosis	Lungs, sinuses, mammalian cells in vitro and in vivo	Pulmonary infections, respiratory failure, disseminated disease	Resistant biofilms, limited understanding of biofilm state in clinical contexts	Treatment with azoles and AmB, surgery to remove infected tissue, especially in severe IA cases
*Malassezia* spp.	Skin and mucosa of humans and animals	Dandruff, seborrheic dermatitis, fungemia, otitis externa, skin infections (veterinary)	Catheters, skin, mucosal surfaces (especially in veterinary settings)	Biofilm formation on catheters; associated with ICU-acquired infections	Up to 2000x resistance in biofilms, especially on medical devices	Combination therapy using ketoconazole and fluconazole. catheter removal and infection site debridement for severe cases. In veterinary cases, additional topical/systemic antifungals
*Trichosporon* spp.	Soil, water, and often related to indwelling devices	Disseminated trichosporonosis, endocarditis, skin infections	Catheters, prosthetic devices, urinary and intravenous catheters	Persistent infections on medical devices; high mortality with biofilm infections	Enhanced antimicrobial resistance, evades immune response due to biofilm structure	Combination therapy with azoles and AmB, removal of biofilm-associated devices and/or surgery in severe cases
*Fusarium* spp.	Soil, decomposing plants, agriculture crops	Keratitis, onychomycosis, invasive fusariosis (e.g., meningitis)	Soil, plant surfaces, human eye, nails, medical devices	Severe, invasive infections, often resistant to treatment	Complex biofilm structure, resistant to multiple antifungal interventions	Keratitis/onychomycosis treatments include natamycin and azoles; systemic infections may require AmB. CNS treatment is complicated by limited drug penetration across the BBB. Some cases may require surgical removal of infected tissue.
*Coccidioides* spp.	Soil in arid regions (Southwest U.S., Central/South America)	Valley fever, chronic coccidioidomycosis, pulmonary infections	Ventriculo-peritoneal shunt tubing, pulmonary tissue	Pulmonary biofilm structures, difficult to diagnose, resistant to treatment	Limited understanding of biofilms, resistant due to immune system evasion strategies	Azoles are used for pulmonary infections. Shunt infections or meningitis, device removal, and high-dose antifungal treatment, often with AmB, are necessary
